# Supplementary material for: The genome sequence of the Anthracite Bee-fly, Anthrax anthrax (Schrank, 1781)
Source: Wellcome Open Res. 2025 Sep 26;10:306. Originally published 2025 Jun 4. [Version 2] doi: 10.12688/wellcomeopenres.24305.2 (PMC12489455; doi:10.12688/wellcomeopenres.24305.2)
Supplement: Peer review history

# Reviewer report: Reviewer response for version 2

**DOI:** 10.21956/wellcomeopenres.27529.r134445

**Published:** 2025-10-01

**Authors:** Arun Arumugaperumal

**Affiliations:** 1 Department of Biotechnology, Rajalakshmi Engineering College, Thandalam 602105, Chennai, Tamil Nadu, India

The authors have carried out the correction. The article can be indexed.

Are sufficient details of methods and materials provided to allow replication by others?

Yes

Is the rationale for creating the dataset(s) clearly described?

Yes

Are the datasets clearly presented in a useable and accessible format?

Yes

Are the protocols appropriate and is the work technically sound?

Yes

Reviewer Expertise:

Bioinformatics; Genomics

I confirm that I have read this submission and believe that I have an appropriate level of expertise to confirm that it is of an acceptable scientific standard.

# Reviewer report: Reviewer response for version 1

**DOI:** 10.21956/wellcomeopenres.26805.r130912

**Published:** 2025-09-18

**Authors:** Arun Arumugaperumal

**Affiliations:** 1 Department of Biotechnology, Rajalakshmi Engineering College, Thandalam 602105, Chennai, Tamil Nadu, India

The data note describes the genome sequence of an Anthracite Bee-fly, *Anthrax anthrax*. The photograph of the specimen used for sequencing is shown in Figure 1. The author has used advanced sequencing technologies to arrive at the chromosome-level genome assembly. The assembly size reported is 334.62 Mb scaffolded into 9 chromosomes, including the XY sex chromosomes. The mitochondrial genomic DNA of size 20.34 kb has also been provided separately. A total of 10,570 protein-coding genes have been identified. These values go hand in hand with those of a similar fly, *Bombylius major*, which is from the same family [1]. The contig and scaffold N50 values were above the threshold values. BUSCO analysis has also revealed that the genome is a near-complete one as evident from the 95.6% completeness. The GC% plot shows more than one distinct blob. As the colour difference of the blob is not deviating much, it can be taken as the absence of any contaminants. The links and accession numbers for raw data and annotation have been provided. The article can be indexed after amending the minor corrections.

 Minor corrections are:

 1. Change '54 coverage' to '54X coverage' in Genome Sequence Report

 2. The quality metrics according to the Earth Biogenome Project guidelines can be mentioned.

Are sufficient details of methods and materials provided to allow replication by others?

Yes

Is the rationale for creating the dataset(s) clearly described?

Yes

Are the datasets clearly presented in a useable and accessible format?

Yes

Are the protocols appropriate and is the work technically sound?

Yes

Reviewer Expertise:

Bioinformatics; Genomics

I confirm that I have read this submission and believe that I have an appropriate level of expertise to confirm that it is of an acceptable scientific standard, however I have significant reservations, as outlined above.

## References

[1] : The genome sequence of the dark-edged bee fly, Bombylius major (Linnaeus, 1758). *Wellcome Open Research* .2023;8: 10.12688/wellcomeopenres.19804.1 10.12688/wellcomeopenres.19804.1 PMC1096400338533437

# Response

**Published:** 2025-09-18

**Authors:** Tree of Life Team Sanger

**Affiliations:** Wellcome Trust Sanger Institute, UK

Thank you for reviewing this data note. We have submitted a revised version addressing your comments.   1) Change “54 coverage” to “54× coverage” in the Genome sequence report.  Corrected. [If the journal prefers ‘X’, we have used “54X”.]   2) The quality metrics according to the Earth BioGenome Project guidelines can be mentioned.  These metrics were already included in version 1 (see “Assembly quality metrics”). We have added brief interpretation of the values.

# Reviewer report: Reviewer response for version 1

**DOI:** 10.21956/wellcomeopenres.26805.r125240

**Published:** 2025-06-25

**Authors:** Anthony Bayega

**Affiliations:** 1 Canada's Michael Smith Genome Sciences Centre (Ringgold ID: 113898), Vancouver, British Columbia, Canada; 2 McGill University, Montreal, Canada

Crowley al. provides a high quality chromosomal-level genome assembly of *Anthrax anthrax*, a commendable effort. My comments follow below:

 1. In Figure 3, the label on the Y-axis which reads “ERR1…” could be changed to something more informative to help the reader make sense of this figure.

 2. In the “Genome annotation report” section, the authors report an average transcript length of 7,275.59 bp. It would be helpful to the reader to know if this is the spliced or unspliced length.

 3. In this particular effort the group has not attempted to decontaminate the assembly of cobionts and other contaminants. This could be highlighted or even explained.

 Overall, the authors provide a good-quality genome and also assign 87.88% of it to chromosomes. Although much work remains to order the scaffolds, fully phase the contigs and scaffolds, and complete the gaps and also structurally and functionally annotate the genome, the current work will indeed be valuable to the whole community. I therefore recommend the indexing of this genome so that this resource becomes widely accessible to the scientific community.

Are sufficient details of methods and materials provided to allow replication by others?

Yes

Is the rationale for creating the dataset(s) clearly described?

Yes

Are the datasets clearly presented in a useable and accessible format?

Yes

Are the protocols appropriate and is the work technically sound?

Yes

Reviewer Expertise:

Human genetics and genomics

I confirm that I have read this submission and believe that I have an appropriate level of expertise to confirm that it is of an acceptable scientific standard.
